# Remote beam output audits: A global assessment of results out of tolerance

**DOI:** 10.1016/j.phro.2018.08.005

**Published:** 2018-09-16

**Authors:** Stephen F. Kry, Christine B. Peterson, Rebecca M. Howell, Joanna Izewska, Jessica Lye, Catharine H. Clark, Mitsuhiro Nakamura, Coen Hurkmans, Paola Alvarez, Andrew Alves, Tomislav Bokulic, David Followill, Pavel Kazantsev, Jessica Lowenstein, Andrea Molineu, Jacob Palmer, Susan A. Smith, Paige Taylor, Paulina Wesolowska, Ivan Williams

**Affiliations:** a Imaging and Radiation Oncology Core, MD Anderson Cancer Center, Houston, USA; b Department of Radiation Physics, MD Anderson Cancer Center, Houston, USA; c Department of Biostatistics, MD Anderson Cancer Center, Houston, USA; d Radiation Dosimetry Services, MD Anderson Cancer Center, Houston, USA; e Dosimetry Laboratory, Dosimetry and Medical Radiation Physics Section, Division of Human Health, International Atomic Energy Agency, Vienna, Austria; f Australian Clinical Dosimetry Service, ARPANSA, Melbourne, Australia; g RadioTherapy Trials Quality Assurance Group, Mount Vernon Cancer Centre, London, UK; h Metrology for Medical Physics, National Physical Laboratory, Teddington, UK; i Department of Medical Physics, Royal Surrey County Hospital, Surrey, UK; j JCOG Division of Medical Physics, Department of Information Technology and Medical Engineering, Human Health Sciences, Graduate School of Medicine, Kyoto University, Japan; k EORTC Radiation Oncology Group, Brussels, Belgium; l Department of Radiation Oncology, Catharina Hospital Eindhoven, The Netherlands

**Keywords:** Global harmonization group, Remote beam output audit, Dosimetry audit, Calibration, QA

## Abstract

**Background and purpose:**

Remote beam output audits, which independently measure an institution’s machine calibration, are a common component of independent radiotherapy peer review. This work reviews the results and trends of these audit results across several organisations and geographical regions.

**Materials and methods:**

Beam output audit results from the Australian Clinical Dosimetry Services, International Atomic Energy Agency, Imaging and Radiation Oncology Core, and Radiation Dosimetry Services were evaluated from 2010 to the present. The rate of audit results outside a ±5% tolerance was evaluated for photon and electron beams as a function of the year of irradiation and nominal beam energy. Additionally, examples of confirmed calibration errors were examined to provide guidance to clinical physicists and auditing bodies.

**Results:**

Of the 210,167 audit results, 1323 (0.63%) were outside of tolerance. There was a clear trend of improved audit performance for more recent dates, and while all photon energies generally showed uniform rates of results out of tolerance, low (6 MeV) and high (≥18 MeV) energy electron beams showed significantly elevated rates. Twenty nine confirmed calibration errors were explored and attributed to a range of issues, such as equipment failures, errors in setup, and errors in performing the clinical reference calibration. Forty-two percent of these confirmed errors were detected during ongoing periodic monitoring, and not at the time of the first audit of the machine.

**Conclusions:**

Remote beam output audits have identified, and continue to identify, numerous and often substantial beam calibration errors.

## 1 Introduction

High quality radiotherapy is critically important for patient outcomes; it also improves the power of clinical trials and thereby improves their effectiveness [1], [2], [3]. High quality radiotherapy requires the accurate calibration of external beam radiotherapy equipment; any error in the clinical reference calibration of a beam is a systematic error that impacts all patients treated with that beam. As such, independent verification of machine output (i.e., a beam output audit) is a standard component of clinical trial quality assurance (QA), and is often conducted as part of good-practice quality assurance. A common approach to such output verification is through a remote audit – i.e., where the dosimeters are mailed to the institution for irradiation.

Numerous QA groups across the world provide independent, remote, beam output audits, and the nature of these programs has been well documented [6], [7], [8], [9], [10], [11], [12]. However, a focused evaluation of audit results outside of tolerance, particularly from a large-scale global perspective, has not previously been performed. The current study therefore presents such an evaluation from remote audits conducted by four QA groups, including identified causes of calibration errors. Such information can provide guidance to the medical physics community about where problems originate, as well as highlighting the value of such remote output verification programmes.

## 2 Materials and methods

### 2.1 Remote beam output audits

Audit results in this study were conducted by four QA groups that are part of the Global Quality Assurance of Radiation Therapy Clinical Trials Harmonisation Group (Global Harmonisation Group [GHG]: https://rtqaharmonization.com/). The GHG works to ensure consistency and coordination of QA efforts. This group is currently comprised of six member groups (who provide quality assurance for clinical trials) and three observer QA groups (who provide radiotherapy quality assurance services not focused on clinical trials) [13]. Remote beam output audits are typically conducted using passive luminescent dosimeters that are mailed to an institution [14]. The institution irradiates them to give a known dose under reference/calibration conditions. These dosimeters are returned and analyzed, and the measured dose is compared to that intended by the institution.

Of the nine groups involved in the GHG, six conduct remote beam output audits. Details about these programmes are shown in Table 1. Although similar, the tolerance for agreement between the measured and stated dose was not identical between groups. Notably, even for a nominal 5% tolerance, some groups round the audit result to 2 decimal places before evaluating (acceptability therefore being ≥0.945 and <1.055) while other groups do not round (acceptability being defined as ≥0.950 and ≤1.050). For consistency and inter-comparability, a ±5%-rounded tolerance (the loosest tolerance) was used in all evaluations in this study (i.e., results outside of tolerance were <0.945 or ≥1.055), even though that did not exactly match the criteria used by some auditing bodies. Results of remote beam output audits were available only for 4 QA groups because not all results were accessible. Therefore only these four groups were evaluated further.

**Table 1:** Methods for conducting remote beam output audits. Details of the dosimeter and dosimetry programme are shown for each QA group in the GHG that performs remote beam output audits.

QA Group	Dosimeter	Frequency	Mandatory audit?	Primary recipients	Uncertainty (%) (k = 1)	Tolerance (±%)	Beams per year (ave)	Key ref(s)
ACDS*	nanoDot OSLD	Every other year	Yes	Australian facilities	Electrons:1.7 Photons: 1.3	Electrons: 5.1 Photons: 3.9	392	[4], [5]
EORTC	Various	When joining a trial if last audit >2 years prior	Yes	European clinical trial participants	Varies-not always known by EORTC	5	356	6
IAEA*	TLD-100	By request	No	Facilities in low/middle income countries	^60^Co: 1.5 X-rays: 1.7	5	623	[7], [8], [9]
IROC*	nanoDot OSLD	Annual	Yes	North American clinical trial participants	1.7	5	16,680	[10], [11]
JCOG**/ANTM	Glass RPLD	Every 3 years	Yes	Japanese facilities	1.1	5	∼500	12
RDS*	TLD-100	By request	No	North American facilities	1.3	5	11,775	11

Abbreviations: ACDS: Australian Clinical Dosimetry Services; EORTC: European Organisation for Research and Treatment of Cancer; IAEA: International Atomic Energy Agency; IROC: Imaging and Radiation Oncology Core; JCOG: Japan Clinical Oncology Group; ANTM: Association for Nuclear Technology in Medicine; RDS: Radiation Dosimetry Services; OSLD: optically stimulated luminescence dosimeter; TLD: thermoluminescent dosimeter; RPLD: radiophotoluminescent dosimeter.

* Beam output audit results evaluated in this study.

** Measurements of the reference output dose for JCOG trials are performed by ANTM for designated cancer centers.

Remote beam output audit results were reviewed from 2010 to the present to examine contemporary machine calibration issues. Minor variations in this time period were allowed to limit the analysis to a single dosimeter: in mid-2010 IROC transitioned from TLD to OSLD; only OSLD results are included. In 2017 the IAEA transitioned from TLD to radiophotoluminescent (RPL) glass dosimeters; only TLD results are included. Additionally, the ACDS has only conducted these audits since 2012.

Individual audit results were excluded from consideration when there were known human errors in the irradiation of the audit dosimeters, e.g., if the institution reported (before any result was issued) that the wrong field size, SSD, or similar had been accidentally used, or when the result had double the expected dose or approximately zero dose (indicating the dosimeters were accidentally irradiated twice and/or not irradiated).

### 2.2 Data analysis

The rate of audit results outside of the ±5% tolerance was compared between the QA groups for each beam type (all, photon, electron). These rates were compared using ANOVA with follow up using pairwise, two-sided, tests including Benjamini-Hochberg corrections for multiple comparisons. These incorporated the binomial nature of the response and a logit link function. The 95% binomial confidence intervals for the rate of results outside of tolerance in each group were computed using the Agresti-Coull method.

The ACDS reported no beams outside a ±5% tolerance, and the IAEA does not audit electron beams, so no further analysis was performed on these data sets. For all other data sets (each QA group and beam type), the out-of-tolerance rates were evaluated both as a function of year of irradiation, and as a function of beam energy.

To assess the rate of results outside tolerance versus the year of irradiation, a generalized linear model was fit for each dataset with a logit link function and a binomial distribution for the rate. This regression model was chosen because it forces the predicted probability of result outside tolerance in future years to remain greater than zero.

To assess the rate of audit results outside tolerance versus beam energy, photon beams were subdivided to include SRS and FFF beams (which did not include Cyberknife or Tomotherapy beams). Cobalt sources included both historical c-arm external beam units as well as modern ViewRay units. All analysis was conducted using ANOVA, assuming a binomial distribution for the rate and a logit link function, and results were evaluated relative to the most common beam energy audited (6 MV for photons and 12 MeV for electrons). Significant ANOVA results were followed up with pairwise tests using the Benjamini-Hochberg correction for multiple comparisons.

When an audit result was outside tolerance, follow up action was undertaken with the goal of understanding and rectifying the cause of the discrepancy. This often involved phone calls, email, or review of calibration worksheets. Most often there was a repeat beam output audit (either because it was mandated or, for voluntary audits, because the institution recognized this as good practice). Often the cause of discrepancy was not ultimately identified (most often because of a lack of communication from the institution). In a small subset of cases, an error was identified and confirmed (e.g., through review of the institution’s calibration worksheets or through verbal confirmation of a calibration error by the institution). These documented calibration errors were reviewed to provide practical information to physicists involved in beam calibration.

## 3 Results

**Fig. 1:**
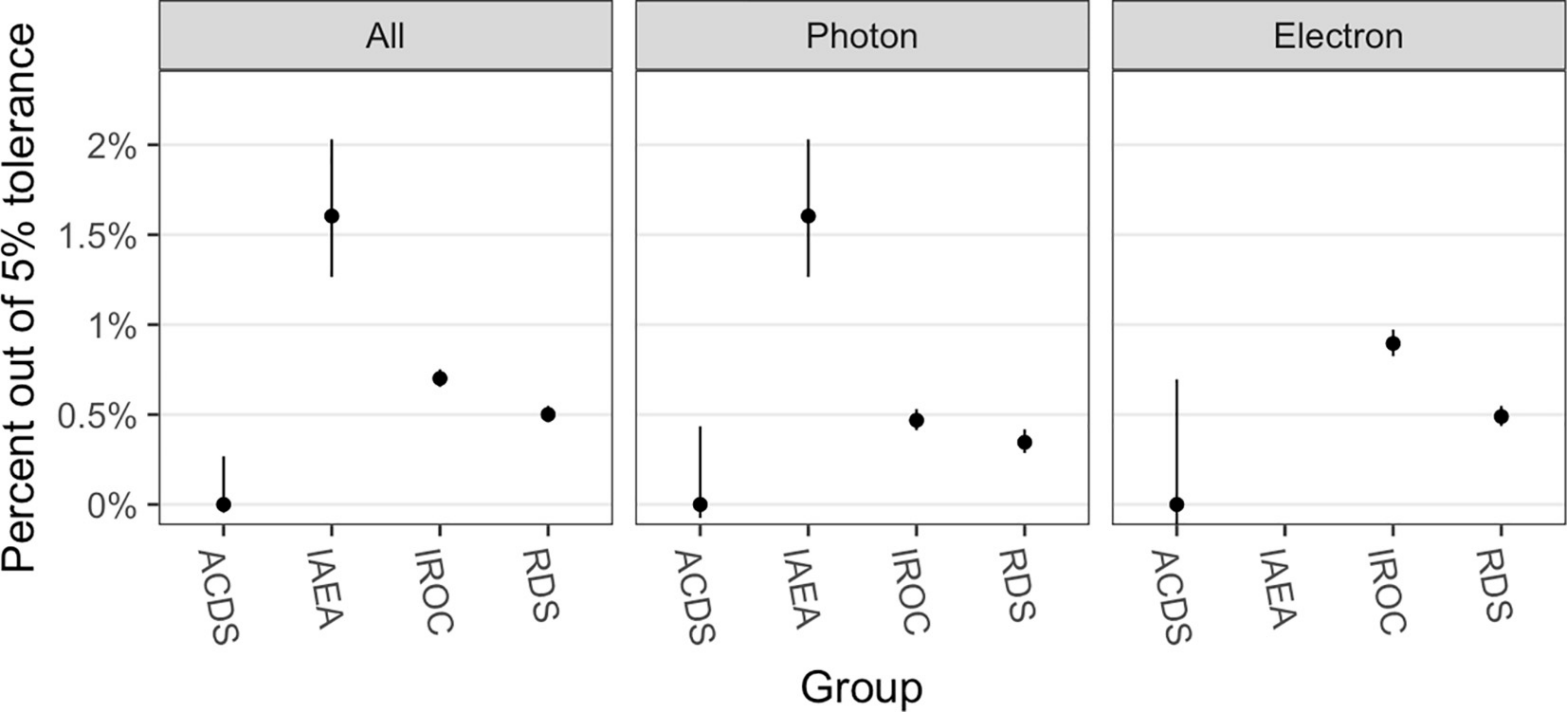
Percentage of remote beam output audit results outside the ±5% tolerance. The IAEA reported the highest percentage while the ACDS reported the lowest. Electron beam results were more likely to be outside of tolerance than photon beams.

A total of 210,167 remote beam output audit results were included in this study, including 114,124 results from IROC, 90,024 results from RDS, 4240 results from the IAEA, and 1779 results from the ACDS. This total was comprised of 87,780 photon beam results, and 122,387 electron beam results. A total of 1,323 results (0.63%) were found to be outside a ±5% tolerance, the distribution of which is shown in Fig. 1 for each QA group. There were significant differences in the rate of audit results outside of tolerance between the QA groups and for each beam type (p ≪ 0.001); significant differences (adjusted p < 0.05) were found between all rates except: ACDS – IROC (photon), ACDS – RDS (photon), and ACDS – RDS (electron). The most pronounced difference was for the IAEA, which had a relatively high rate of results outside of tolerance (1.6%). The ACDS had the lowest rate of results outside of tolerance: 0%. IROC and RDS had intermediate rates.

The rate of audit results outside of tolerance for photon versus electron beams indicated that there were significantly more results outside of tolerance for the electron beams than for photon beams (p ≪ 0.001 IROC, p = 0.002 for RDS; two-sided test for equality of proportions).

**Fig. 2:**
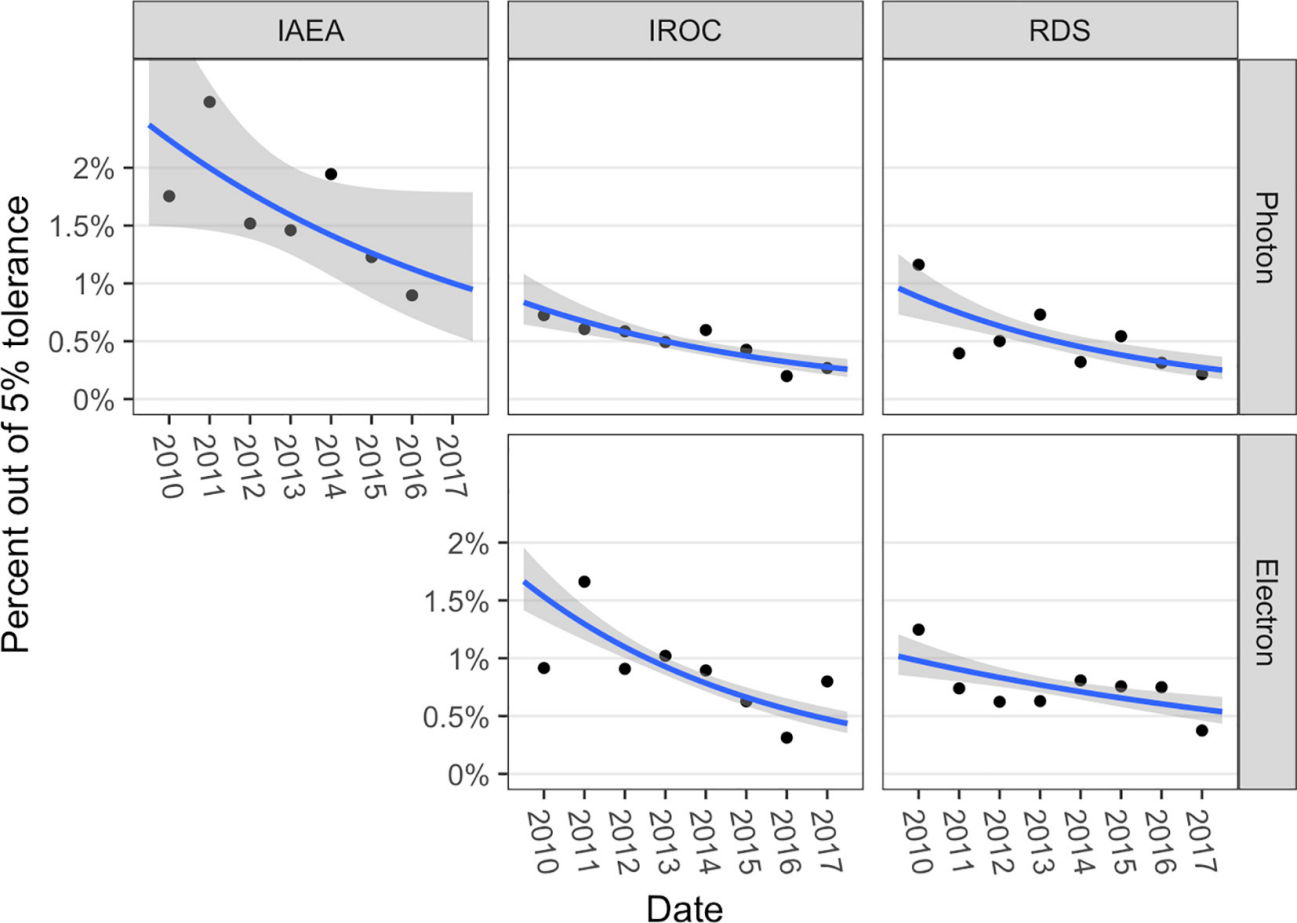
Percent of remote beam output audit results outside the ±5% tolerance as a function of audit year. The blue line is the fitted regression line and the grey areas represent the 95% confidence intervals. The grey area is larger for the IAEA results because of the smaller number of results. Audit results have shown significant improvement with time. (For interpretation of the references to colour in this figure legend, the reader is referred to the web version of this article.)

The audit results as a function of audit year are shown in Fig. 2. All groups showed a clear trend for fewer results outside of tolerance in more recent years. While the IAEA trend did not achieve statistical significance (p = 0.07), all other trends were highly significant (p ≪ 0.001).

**Fig. 3:**
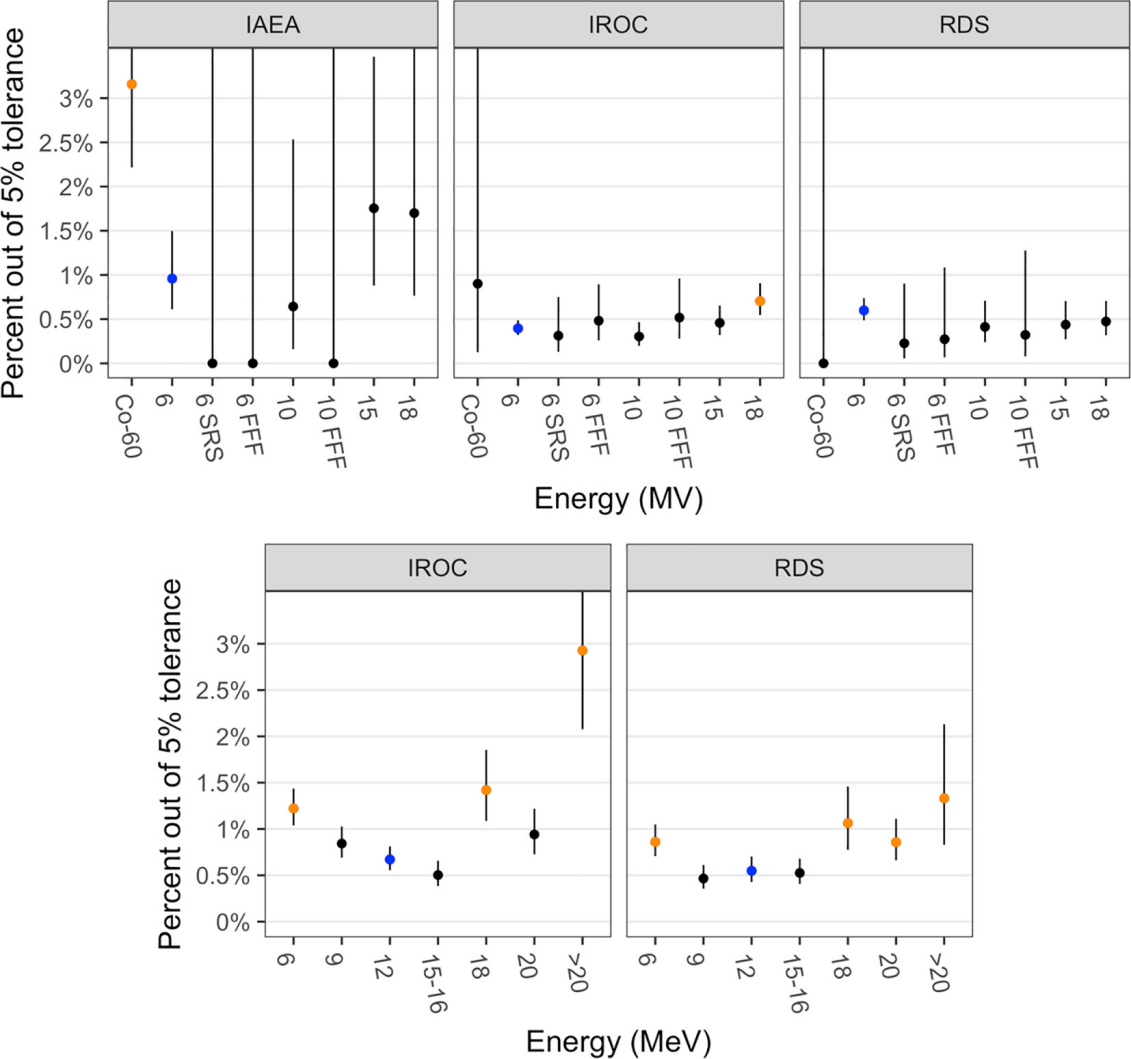
Percent of remote beam output audit results outside the ±5% tolerance as a function of beam energy (MV for X-rays, MeV for electrons). The blue dot is the most common energy (6 MV and 12 MeV) against which other energies were compared. Orange dots are those significantly different from the most common. (For interpretation of the references to colour in this figure legend, the reader is referred to the web version of this article.)

The rate of audit results being outside tolerance versus beam energy are shown in Fig. 3. For photon beams, there was little difference between energies except for cobalt beams audited by the IAEA, which showed a highly elevated rate. Electron beams, in contrast, did not show uniform audit rates by energy. Both IROC and RDS observed that higher and lower electron beam energies were more prone to audit results being outside tolerance.

**Fig. 4:**
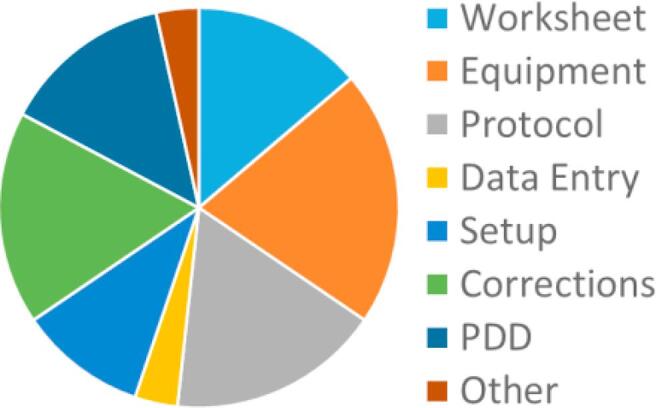
Primary cause of confirmed calibration errors.

From beam output audits, a calibration error was confirmed and documented in detail at 29 institutions. These errors impacted the calibration of 93 different beams. Most of the results (22 out of 29) presented here were documented by IROC. Of these 29 errors, seven were in excess of 8%. Errors were found with a variety of different causes, including errors in the following: worksheet (incorrect calculation implemented in the calibration worksheet, often not multiplying the factors correctly or incorrectly copying factors from one energy to another); equipment (broken or inappropriate equipment, or instability of equipment used in calibration); protocol (failure to follow protocol instructions, most often failure to apply any percent depth dose (PDD) correction, particularly for electron beams to relate output at *d_ref_* to that at *d_max_*); incorrectly determining the PDD to relate output at the measurement point to the reference point (i.e., correctly following the protocol to apply this PDD correction, but incorrectly determining what that correction should be, e.g., using percent ionization instead of percent dose); data entry (incorrect numbers (e.g., *k_e_*) used in the calibration); setup (incorrect setup during calibration); incorrect determination of correction factors (most often incorrect determination of pressure and therefore *k_tp_*, or *k_Q_*). The relative frequency of these different causes of error are shown in Fig. 4, illustrating the relatively even distribution of causes of error.

Other results were also apparent from these 29 confirmed calibration errors. First, the results were relatively evenly divided into whether the errors were systematic and affected all beams or whether the error affected only a single (or at most two) beams. Thirteen confirmed errors affected all beams on the radiotherapy unit (including three cobalt units with only one beam). These errors often involved malfunctioning or misused equipment, including several errors relating to the barometer. Three confirmed errors affected only the photon beams or only the electron beams, but not both. The remaining 13 errors affected only one or two beams. A second finding from these results was that eight errors (28%) were identified and resolved without a result actually exceeding the ±5% tolerance. These errors were identified because a beam was at the edge of tolerance and follow-up was undertaken even though the audit was passed, or because a trend was observed in a beam’s output during repeated, periodic auditing (e.g., consistently 3–5% high). Finally, it was noted that in 12 cases (41%), the error was not detected on the first audit of that beam, but rather was found during subsequent/repeated audits of the beam (e.g., by groups that perform periodic/annual monitoring). In four of these 12 cases, the error was detected as a trend in the output (i.e., being within tolerance but consistently high or low, year after year); in the other eight cases the beam was originally calibrated correctly but then an error was introduced into the calibration.

## 4 Discussion

This work reviewed the findings of four auditing groups for the recent history of their remote beam output audit results. Because the EORTC audits were performed by several different auditing bodies, including IROC, and the reports were provided by the institutions, it was not possible to collect all results, or incorporate them, into the current study. Similar obstacles prevented audit data from JCOG from being included in the current analysis.

Overall, the frequency that any given beam would be out of tolerance was low (0.63% on average). However, the rate with which an institution had at least one beam outside of tolerance is much higher because institutions typically maintain numerous beams. For example, IROC audits (which include all photon and three electron beams per machine each year) found that 4.5% of institutions (on average) had at least one beam out of tolerance during any given year; over the ∼8 years of this study, 19% of institutions had at least one beam outside of tolerance. Considered in this manner, these audits yield a large number of results that require follow-up. Offsetting the benefit of identifying these results is the cost associated with conducting such audits; a cost-benefit analysis for remote beam output audits is planned for a future study.

This study found four main relationships. First, there were differences in the rate of audit results being outside of tolerance between audit groups, most notably that audits conducted by the IAEA were more likely to be outside tolerance than for other groups (particularly for cobalt units). This elevated result may be understandable given that the IAEA uniquely services low and middle income countries that often struggle to obtain sufficient radiotherapy resources. Second, electron beams were more often found outside of tolerance than photon beams. As indicated in specific examples of confirmed calibration errors, electron beam calibration protocols involve more steps that are less uniform across energies than for photon beams, include a conversion of ionization to dose, involve steeper dose gradients, and even include erroneous calibration values (e.g., some *k_ecal_* values in TG-51) [15], [16]. All of these issues contribute to higher rates of audit results outside of tolerance for electron beams. Third, the rate of results out of tolerance has been decreasing over the past several years. The reason for this improvement is not obvious; there have been no major changes in calibration protocols during this time period and these audits have been consistent and ongoing for decades. Thus, this improvement suggests an improved implementation of the protocols, possibly through increased familiarity, or increased standards and consistency in the training of medical physicists. The feedback and guidance on calibration procedures provided through these audit programmes may also be contributing to the long-term increased performance of the radiotherapy community. Fourth, there were differences in rate of audit results outside tolerance as a function of beam energy; rates were elevated for low and high energy electron beams. It is possible that the higher dose gradients of low energy electron beams make them more susceptible to calibration errors because the measurements are more sensitive to equipment selection and positioning errors. Alternatively, low energy electron beams are often calibrated with parallel plate chambers, but these are associated with higher uncertainty in the calibration of the beam [17]. High energy electron beams were apparently prone to error in the determination and application of the PDD correction to move to *d_ref_* (*z_ref_*).

It was found that calibration errors originated from a wide range of sources, highlighting the challenges of machine calibration in terms of the number of processes that can go awry. Such calibration errors could impact a single beam, multiple beams, or all beams at a facility. Additionally, it was found to be relatively common that a calibration error was introduced into a historically accurate output. This indicates that there is substantial value to routine and/or periodic auditing of radiotherapy beams. Finally, although the tolerance of this audit is relatively high (±5%), it is possible to detect errors that are smaller (although this is more challenging given the uncertainty in the dosimeters).

A beam output audit result outside of tolerance does not necessarily mean that there is an error in the beam calibration. There are three possible causes of such a result. First, the calibration of the machine could be perfect, yet an out-of-tolerance result could arise from statistical noise in the dosimetry system (∼1.5% coefficient of variation; Table 1). Assuming a normal distribution of measurement uncertainty, 50 results would be expected to be outside of tolerance because of noise in the system, corresponding to <4% of the 1,326 reported results outside of tolerance. Second, there could be an incorrect irradiation of the dosimeter during the audit or incorrect reporting of the external beam calibration, i.e., an error in the institution’s performance of the audit (e.g., using the wrong energy, field size, or SSD when irradiating the dosimeter, or reporting the calibration condition to be SSD when it is SAD). Such errors definitely happen, although their impact on the results presented is minimized to the extent possible: if an irradiation error is noted and reported, the audit results are adjusted to account for it (if possible) or rejected from being reported (if not). Either way, the result is expunged from the records and excluded from this study. Nevertheless, many such errors are unidentified and therefore captured as a result out of tolerance. It is hard to estimate the number of such events because, by their nature, they are unknown. The third, and critical, reason a result may be out of tolerance is because of an error in the calibration of the radiotherapy unit. Fig. 4 highlights many cases where such problems have been identified. Unfortunately, this list is only a small subset of the audit results that are outside of tolerance. In most cases where there is a result out of tolerance, the cause and resolution of this issue is not communicated to the QA group; this is a major limitation of the remote nature of this audit.

While the true calibration error rate is unknown from this data, the findings of this study can be compared to other sources. The EORTC found 13 beams outside of a 5% tolerance (nine electron and four photon) of out of 1790 photon and 1366 electron beams audited between 2005 and 2014 [6]. This corresponded to an overall rate of 0.41%, very similar to the overall rate of 0.63% in the current study. In addition, a recent review of calibration errors identified and confirmed using Farmer-type chambers during on-site audits was recently published by IROC (based on 1,020 audited linacs between 2000 and the present) [18]. Twenty percent of institutions had an error ≥ 4% in at least one electron beam calibration and 8% had an error ≥ 4% in at least one photon beam calibration. This error rate is similar if not higher than that from the current study where 4.5% of institutions (remote audited by IROC) that had a result outside of the ±5% tolerance. These on-site findings indicate that a large number of the unconfirmed remote audit deviations could very likely be legitimate calibration errors. The legitimacy and relevance of the remote audit findings from the current study are also supported by trends seen in the on-site audit data: that electron beams were more likely out of tolerance than photon beams, that high and low energy electron beams were more likely to be outside of tolerance than intermediate energy electron beams, and that electron beam calibration errors were becoming less common with time [18]. These trends were the same as those found from the remote output audit programmes. In addition to this, an improvement in calibration accuracy with time was also found during on-site audits throughout the UK [19], where improvements in both photon and electron beam calibrations were identified during the audit period as well as in comparison to earlier audits in the UK [20], [21]. However, these UK on-site audit results did not find differences between photon and electron beams, or between different electron beam energies. This could reflect differences in the calibration protocol implemented in the UK, but could also simply reflect that this study lacked the power to detect the relatively small differences, including only 81 photon and 98 electron beams in the audit.

In conclusion, remote beam output audits identified a number of errors in external beam radiotherapy machine calibration across the world. Major errors were seen in almost all parts of the calibration process, including following the calibration protocol, calculation and worksheet errors, equipment problems, and errors in calibration setup. Some errors, such as using incorrect calibration worksheets or setting up the calibration condition incorrectly, can be substantially mitigated through good training and attentive calibration. Other errors, such as failures of equipment, can be much more insidious. Regardless, the value of independent peer review of beam calibration is high, including (but not limited to) remote beam output audits as demonstrated in this study.

## 5 Conflict of interest

No conflict to declare.
